# Addressing tobacco in Australian alcohol and other drug treatment settings: a cross-sectional survey of staff attitudes and perceived barriers

**DOI:** 10.1186/s13011-017-0106-5

**Published:** 2017-05-02

**Authors:** Eliza Skelton, Flora Tzelepis, Anthony Shakeshaft, Ashleigh Guillaumier, Adrian Dunlop, Sam McCrabb, Kerrin Palazzi, Billie Bonevski

**Affiliations:** 1 The University of Newcastle, Faculty of Health and Medicine, School of Medicine and Public Health, 1 University Drive, Callaghan, NSW 2308 Australia; 2 Hunter New England Local Health District, Hunter New England Population Health, Booth Building, Longworth Avenue, Wallsend, NSW 2287 Australia; 3 Hunter Medical Research Institute (HMRI), 1 Kookaburra Circuit, New Lambton Heights, NSW 2305 Australia; 4 The University of New South Wales, National Drug and Alcohol Research Centre, 22-32 King Street, Randwick, NSW 2031 Australia; 5 Hunter New England Local Health District, Newcastle Community Health Centre, 670 Hunter Street, Newcastle West, NSW 2302 Australia

**Keywords:** Alcohol and other drugs, Substance abuse treatment, Smoking cessation, Tobacco, Cross-sectional survey, Attitudes, Barriers

## Abstract

**Background:**

Within alcohol and other drug (AOD) services, staff attitudes and beliefs are important influences determining provision of smoking cessation care. This study of AOD staff aimed to examine: a) current attitudes toward smoking cessation care; b) service and staff characteristics associated with unsupportive smoking cessation care attitudes, and c) perceived barriers to providing smoking cessation care.

**Methods:**

Between July-October 2014, 506 staff from 31 Australian AOD services completed an online cross-sectional survey which assessed agreement with 6 attitudinal statements (supportive and unsupportive) and 10 perceived barriers to smoking cessation care in the AOD setting. Logistic regressions examined service (sector) and staff (age, gender, smoking status and number of years in AOD field) characteristics associated with unsupportive smoking cessation care attitudes.

**Results:**

A large proportion agreed with supportive statements: Smoking cessation care should be part of usual care (87%), smoking cessation care is as important as counselling about other drugs (72%) and staff have the organisational support to provide smoking cessation care (58%). Some respondents agreed with unsupportive statements: AOD clients are not interested in addressing their smoking (40%), increasing smoking restrictions would lead to client aggression (23%), smoking is a personal choice and it is not the service’s role to interfere (16%). Respondents from non-government managed services, current tobacco smokers (compared to ex-smokers) and those with less AOD experience had higher odds of agreeing with unsupportive smoking cessation care statements. The most frequently identified barriers to providing smoking cessation care were: client inability to afford cessation medicines, insufficient funding and lack of a coordinated treatment approach (all 61%).

**Conclusions:**

Overall, staff hold largely supportive smoking cessation care attitudes but perceive a large number of barriers to providing smoking cessation care.

## Background

Tobacco smoking rates in alcohol and other drug (AOD) treatment services are as high as 87% [1, 2]. AOD clients are more likely to die from tobacco-related diseases [3–5] than from their other substance abuse. Recognising the importance of reducing tobacco smoking harms in clients engaged in AOD treatment, national clinical practice guidelines in the US [6], UK [7, 8] and Australia [9] recommend the delivery of evidenced-based smoking cessation care as part of usual care. However, current smoking cessation care practices in the AOD setting falls short of clinical practice guidelines [10, 11].

Staff attitudes towards smoking cessation care are important determinants of provision of care [12]. The Theory of Planned Behaviour identifies that a person’s attitudes, social norms and behavioural control influences behavioural intention which leads to behaviour [13]. Previously identified unsupportive AOD staff attitudes to smoking cessation care include: beliefs about smoking cessation compromising treatment goals for other substances [14]; tobacco dependence being a lower treatment priority than addiction to other substances [15]; clients not willing or interested in addressing their tobacco smoking [16]; and the belief that aggression towards staff will increase if tobacco smoking is addressed [14].

AOD staff attitudes are influenced by individual and service-related characteristics [12, 17, 18]. AOD staff identifying as never and ex-smokers are significantly more likely than smokers to be in favour of smoking cessation care being delivered concurrently with treatment for other addictions [19]. AOD staff with more nicotine addiction training are significantly more likely to agree that counselling is beneficial for client smoking cessation compared to professionals with less training [19]. Furthermore, AOD staff in a clinical role who are certified or licensed hold less favourable attitudes about treating tobacco smoking compared to non-clinicians [12]. Service-related characteristics such as having a written total ban smoking policy [20] and written smoking cessation care protocols [11] have been associated with supportive staff attitudes towards smoking cessation care. However whether other service-related characteristics such as ownership (e.g. government and non-government services) are associated with staff smoking cessation care attitudes is unexplored. Further, very few studies have included individual and service-related characteristics in models examining AOD staff smoking cessation care attitudes [12, 18].

Barriers to the implementation of a health practice can be structural (e.g. physical environment) [15], cultural (e.g. acceptance of smoking) [21], resource (e.g. little or no smoking cessation specific training) [22] or individual (e.g. staff smoking status) [14]. While barriers to providing smoking cessation care in the AOD setting are well documented [14, 15, 23, 24] only two studies have sought to determine which barriers are perceived more important than others [25, 26]. However these studies did not examine potentially important cultural (e.g. smoking is not core business) and individual (e.g. client inability to afford smoking cessation aids barriers. To address this gap in the literature, research is needed that investigates such barriers and identifies which of these barriers most inhibits provision of smoking cessation care.

Given that AOD staff practices may be influenced by their attitudes it is essential to understand current beliefs, investigate previously unexplored characteristics associated with these beliefs and additional potential barriers that may inhibit care delivery to ensure that effective interventions are designed. This study of AOD staff aims to examine the: I) current attitudes toward smoking cessation care; II) service and staff characteristics associated with unsupportive smoking cessation care attitudes; and III) perceived barriers to providing smoking cessation care.

## Methods

### Study design

An online cross-sectional survey with staff from 31 Australian AOD treatment services conducted in July to October 2014. A telephone interview was also conducted with a site contact at each service to gather service-related data.

### Setting

Thirty-one AOD services in four states and territories of Australia: New South Wales (NSW), Australian Capital Territory (ACT), Queensland (Qld) and South Australia (SA) were included in this survey. Government services were recruited through key contacts such as directors of health services while non-government services were invited through peak bodies who promoted the research in their newsletters. Eligibility requirements included they provide services with face-to-face client contact, and that they see at least 50 clients per year. Of 32 eligible services that expressed interest in participating, 31 completed the study. An audit of written tobacco smoking policies by the research team revealed that 19 (61%)services had total bans, 11 (36%) had partial bans and 1 (3%) did not have a written smoking policy [27].

### Participants

Eligible participants were all current staff members (including nursing, administration, security) at the 31 participating treatment services and were employed in a voluntary, casual, part-time or full-time position.

### Procedure

The research team communicated with one staff member as the site contact from each service. The research team sent the site contact an invitation email containing the participant information letter and the hyperlink to the online survey for distribution to staff. Survey completion constituted consent. Weekly reminder emails (one per week for three weeks) were sent to all staff members. Ethical approval was obtained through the Hunter New England Local Health District’s Human Research Ethics Committee (HREC), ACT Health HREC, SA Health HREC and the University of Newcastle HREC.

## Measures

### Attitudes

Six statements about smoking cessation care provision were presented in random order: 1) A comprehensive range of smoking cessation treatments should be part of normal care in this service; 2) Smoking cessation counselling is as important as counselling about other drugs for clients of this service; 3) Our staff have the organisational support to provide smoking cessation treatments to clients; 4) Increasing restrictions on smoking in this service would increase client aggression towards staff; 5) Smoking is a personal choice and it is not this service’s role to interfere; 6) Most drug and alcohol clients who smoke are not interested in doing anything about their smoking. A five-point response scale was used ranging from “strongly disagree” to “strongly agree”.

### Barriers to the provision of smoking cessation care to clients

Ten perceived barriers to providing smoking cessation care to clients were examined by asking respondents to rate on a four point Likert scale from 1 = Very important to 4 = Not at all important, “How much of a barrier to the provision of smoking cessation care for clients in your organisation are the following”: 1) There is a lack of staff time to provide smoking cessation support; 2) There is a lack of a coordinated staff approach in providing smoking cessation care; 3) There is a lack of staff training in smoking counselling; 4) Staff are uncertain about effective smoking cessation interventions; 5) Clients spend too little time at the organisation to be counselled about their smoking; 6) Clients are unable to afford smoking cessation medicines e.g. Nicotine Replacement Therapy (NRT); 7) Clients are unable to access smoking cessation services once back in the community e.g. Smoking Cessation Programs; 8) There is a concern that there could be a potential impact of providing this support to clients and that it will affect their other drug issues; 9) There is a lack of funding to the organisation to address smoking; and 10) Addressing smoking is not regarded as part of core business for the organisation.

### Staff characteristics

Gender, age, highest qualification, smoking status, current client contact, employment status, years employed at current AOD service, years employed in AOD field and work role were collected.

### Service characteristics

A research team member telephoned the site contact and collected information on the following: government-managed or non-government managed service, treatment program and location (major city, inner/outer regional area) based on the Accessibility/Remoteness Index of Australia (ARIA+) [28, 29]. Primary substances addressed by the AOD service were also assessed by asking staff in the online survey. Respondents could select as many responses as applied from a list of substances (any psychotic drug, alcohol, benzodiazepines and other sedatives, cannabis, heroin and other opioids, psychostimulants incl. amphetamines, any other illegal drug).

### Statistical analysis

Staff and service characteristics are presented by frequencies and percentages for categorical variables and means (standard deviation [SD]) or medians interquartile range [IQR]) for continuous variables depending on the distribution. Attitude statements were grouped into three categories: strongly disagree/disagree, neither agree nor disagree, strongly agree/agree with frequencies and percentages reported. Barriers to the provision of smoking cessation care were grouped into quite/very important vs a little important/not important with frequencies and percentages for each barrier reported.

Binary logistic regression was used to examine service and staff characteristics associated with strong agreement/agreement on the three unsupportive smoking cessation care statements. Variables included in the logistic regressions were selected a priori and included factors previously explored with attitudes towards smoking cessation care in the AOD setting (gender [12], smoking status [14], years in AOD field [12]) and those unexplored (government or non-government managed service, role, age). Presence of an organisational tobacco smoking policy was included in the preliminary binary logistic regression models however collinearity was indicated with government or non-government managed service and therefore was removed. Adjusted estimates or odds ratios, with 95% confidence intervals and *p*-values are presented for variables in the model. Collinearity of variables was checked using variance inflation factors (VIFs). Correlation within individuals from the same service was examined by fitting a model general estimating equation (GEE) with and without a repeated statement for service and examining model fit. If model fit was not improved (Quasi-information criterion [QIC] >4 points less) then logistic regression without clustering was used. Significance was determined at *p* <0.05. SAS 9.4 (SAS Institute Inc., Cary, NC, USA) was used for analyses.

## Results

### Service characteristics

Most services were: located within a major city (77%), government-managed (58%). A variety of AOD treatment program types were included: residential rehabilitation/therapeutic community (*n* = 13, 42%), out-patient counselling (*n* = 9, 29%), opiate treatment/methadone maintenance (*n* = 4, 13%), specialist detoxification unit (*n* = 2, 6%), and other- harm minimisation (*n* = 2, 6%), other- area health (*n* = 1, 3%). Staff reported that the most frequently addressed substances by their service were: Heroin and other opioids (82%), Alcohol (82%) and Cannabis (79%).

### Staff characteristics

Overall, 506 respondents participated from 882 invitations (57% response rate). Participants had a mean age of 45 years (SD = 12), 70% were female and 63% were university educated. Sixteen per-cent identified in a management role and the most frequently reported staff roles were: Nurse (25%), Case-worker (18%) and Counsellor (11%). Most staff (*n* = 378, 76%) indicated that they had current client contact and that providing treatment was part of their usual duties. About two-fifths of respondents were ex-smokers (43%), 32% were never -smokers and 25% were daily or occasional smokers (see Table 1).

**Table 1:** Staff characteristics^a^

Characteristic	n^b^	%
Gender		
Female	322	70
Male	138	30
Age in years (mean, SD)^c^	45(12)	
Highest work qualification		
School certificate/Higher school certificate	18	5
TAFE^d^ certificate/diploma	118	32
University undergraduate/post graduate degree	233	63
Smoking status		
Ex-smoker	188	43
Never-smoker	142	32
Daily/Occasional smoker	108	25
Role		
Manager	81	16
Nurse	126	25
Caseworker	91	18
Counsellor	57	11
Administration	48	9.6
Psychologist	20	4
Social worker	18	3.6
Medical Practitioner (specialist/generalist)	15	3
Health Educator	14	2.8
Researcher	7	1.4
Volunteer	4	0.8
Pharmacist	2	0.4
Other	13	2.6
Employment status		
Full-time	307	62
Part-time	155	32
Casual	26	5.3
Volunteer	3	0.6
Number of years at organisation		
< 1 year	62	13
1–3 years	127	26
4–6 years	106	22
7–9 years	66	14
≥ 10 years	127	26
Number of years in AOD field		
< 1 year	39	8
1–3 years	101	21
4–6 years	88	18
7–9 years	66	14
≥ 10 years	193	40

^a^Descriptive statistics are presented by counts and percentages for categorical variables

^b^may not equal 506 for staff due to missing data

^c^Age is a continuous variable that was found to be normally distributed, the mean and standard deviation is presented

^d^TAFE: Technical and Further Education

### Attitudes towards smoking cessation care

The majority of staff agreed that a comprehensive range of smoking cessation treatments should be part of usual care (87%), that smoking cessation counselling is as important as counselling about other drugs (72%) and that staff have the organisational support to provide smoking cessation treatments to clients (58%). While a minority agreed that most drug and alcohol clients who smoke are not interested in doing anything about their tobacco smoking (40%), that increasing restrictions on tobacco smoking would increase client aggression towards staff (23%) and that smoking is a personal choice and that it is not the service’s role to interfere (16%) (Table 2).

**Table 2:** Level of agreement to attitudinal statements regarding the provision of smoking cessation care by staff^a^

Statement	Strongly disagree/disagree		Neither agree nor disagree		Strongly agree/agree	
	n	%	n	%	n	%
Supportive						
A comprehensive range of smoking cessation treatments should be part of usual care	12	3	43	10	388	87
Smoking cessation counselling is as important as counselling about other drugs for clients of this service	52	13	73	16	318	72
Our staff have the organisational support to provide smoking cessation treatments to clients	77	18	110	24	256	58
Unsupportive						
Most drug and alcohol clients who smoke are not interested in doing anything about their smoking	155	35	110	25	178	40
Increasing restrictions on smoking in this service would increase client aggression towards staff	220	50	122	28	101	23
Smoking is a personal choice and it is not this service’s role to interfere	245	55	126	28	72	16

^a^Attitudinal statements were rated on a 5-point likert-type scale. For analysis purposes responses were grouped as strongly disagree/disagree, neither agree nor disagree, strongly agree/agree. Counts and percentages are presented

### Staff and service characteristics associated with unsupportive smoking cessation care attitudes

The results of the multivariate regressions are presented in Table 3. AOD staff who identified as current smokers compared to ex-smokers and those with <1 years, 4–6 years and 7–9 years compared to those with ≥10 years of employment in the AOD field had higher odds of agreeing with the statement “*most drug and alcohol clients who smoke are not interested in doing anything about their smoking”.* Staff from non-government services compared to government services and staff who were current smokers compared to ex-smokers had higher odds of agreeing with the statement “*increasing restrictions on smoking in this organisation would increase client aggression towards staff”.* Staff identifying as current smokers had higher odds of agreeing with the statement “*Smoking is a personal choice and it is not this organisation’s role to interfere*” compared to ex-smokers.

**Table 3:** Service and staff characteristics associated with unsupportive attitudes towards smoking cessation care^a^

Statement	OR	95%CIs	*P*
Most drug and alcohol clients who smoke are not interested in doing anything about their smoking			
Smoking status (reference: ex-smoker)			0.010
Current-smoker	2.61	1.37,4.99	0.004
Neversmoker	1.71	1.00,2.90	0.049
Number of years in the AOD field (reference: 10+ years)			0.005
< 1 year	8.62	2.50,29.71	0.001
1–3 years	1.70	0.83,3.49	0.150
4–6 years	2.22	1.07,4.61	0.032
7–9 years	2.59	1.17,5.70	0.018
Increasing restrictions on smoking in this organisation would increase client aggression towards staff			
Non-government managed service (reference: government managed service)	2.81	1.59,4.98	<0.001
Smoking status (reference: ex-smoker)			0.008
Current-smoker	3.03	1.50,6.12	0.002
Never smoker	1.80	0.95,3.42	0.072
Smoking is a personal choice and it is not this organisation’s role to interfere			
Smoking status (reference: ex-smoker)			0.016
Current-smoker	2.95	1.41,6.18	0.004
Never smoker	1.76	0.87,3.53	0.114

^a^Binary logistic regression was used to examine the staff and service characteristics associated with agreement to each of the three unsupportive attitude statements. Variables shown in the table are those that showed a significant association to each statement. Each model included service ownership, gender, age, smoking status, number of years in the AOD field

### Barriers to the provision of smoking cessation care to clients

The most common barriers identified as very/quite important were: client inability to afford smoking cessation medicines (61%), lack of funding to the organisation to address client tobacco smoking (61%), lack of a coordinated staff approach (61%) and lack of staff training in smoking counselling (60%) (Table 4).

**Table 4:** Staff-reported barriers to the provision of smoking cessation care to AOD clients^a^

Barrier	Very/Quite Important	
	n^b^	%
Clients are unable to afford smoking cessation medicines	279	61
Lack of funding to the organisation to address client tobacco smoking	278	61
Lack of a coordinated staff approach	275	61
Lack of staff training in smoking counselling	271	60
Lack of staff time to provide smoking cessation support	235	52
Staff are uncertain about effective smoking cessation interventions^a^	175	51
Clients are unable to access smoking cessation services once back in the community	218	48
Addressing smoking is not regarded as part of core business for the organisation	206	45
There could be a potential impact of providing this support to clients and that it will affect their other drug issues	187	41
Clients spend too little time at the organisation to be counselled about their smoking	173	38

^a^Perceived barriers to providing smoking cessation care were rated on a 4-point likert-type scale. For analysis purposes responses were grouped as: very important/quite important, a little important/not important. Counts and percentages are presented for barriers rated as very important/quit important

^b^Presented to *n* = 343 as the survey item was introduced later

## Discussion

Overall, AOD staff hold largely supportive smoking cessation care attitudes including the beliefs that smoking cessation care should be part of usual care and that smoking cessation counselling is as important as counselling about other drugs. Staff attitudes were influenced by their own smoking status, service ownership and the number of years in the AOD field. Despite AOD staff’s supportive attitudes important barriers to the provision of smoking cessation care were identified.

Our findings highlight the significant progress towards a culture supportive of smoking cessation care in AOD settings. The first, and only other, Australian study of AOD staff SCC attitudes was conducted in 2000-1 with one manager and one staff member (*n* = 417) from 260 services across Australia completing a paper-based survey about their personal attitudes and practices in relation to the provision of SCC [25]. In Walsh and colleagues study 63% agreed that a comprehensive range of smoking cessation interventions should be part of their service and 52% agreed that smoking cessation counselling is as important as counselling about other drugs for clients of the service [25]. Comparatively, in our study 87 and 72% of staff agreed with these same statements, respectively. There also appears to be a reduction in the belief among AOD staff that clients are disinterested in addressing their tobacco smoking (66% agreed in Walsh and colleagues study vs. 40% in the current study). It is promising that staff are recognising client smoking cessation interest which may lead to staff being more likely to discuss the client’s quit intentions or potential treatment options.

Like prior research [12, 19], AOD staff who are current tobacco smokers were more likely to agree with unsupportive smoking cessation care statements. A recent qualitative study with AOD staff proposed that this may be due to fear of job security [30]. Staff who were current smokers cited the implementation of smoking cessation care as usual care may prompt services to introduce a non-smoker eligibility criteria for employees and that in other services treating client smoking, staff are required to sign written contracts agreeing not to smoke or their employment will be terminated [30]. Staff who smoke tobacco may find it difficult to quit smoking and therefore may not feel well-equipped to address client tobacco smoking.

This is the first study to examine service ownership as a factor associated with AOD staff smoking cessation care attitudes. Our results suggest that non-government staff are more likely to believe that increasing restrictions on tobacco smoking would lead to aggression towards staff. In Australia, government-managed services are required by state and territory law to be smoke-free environments [31]. Evidence from the broader AOD literature suggests that the implementation of total ban smoking policies does not increase client aggression towards staff or lead to other aversive outcomes and that services with smoke-free policy have a workplace culture more favourable towards smoking cessation care [20, 32]. It is possible that AOD staff from government-managed services are less likely to agree with this statement as they are already working in a restricted tobacco smoking environment and may not have experienced client aggression.

Unlike another study [12] examining the number of years employed in the AOD field as a factor associated with AOD staff smoking cessation care attitudes, our findings revealed a significant relationship. Although only speculative, it is possible that those with less time in the field would have had less treatment experience, less opportunity to receive further smoking cessation care training and would be less likely to provide smoking cessation care or to recognise that clients expect their tobacco smoking to be treated. Current evidence suggests that AOD staff with more smoking cessation education are more likely to hold supportive smoking cessation care attitudes and subsequently are more likely to address their clients’ tobacco smoking [19].

Our study is one of the few to ask respondents to attribute the level of importance of well-documented barriers [25, 26]. Six of the ten barriers were identified as very/quite important by the majority and highlighted that AOD staff experience a number of barriers when attempting to provide smoking cessation care. The proportion of staff reported barriers are similar to those reported in a study conducted more than a decade ago: i.e. lack of coordinated staff approach (our 61% compared to 65%), lack of staff training in smoking counselling (our 60% compared to 64%) and lack of staff time (our 52% compared to 55%) [25]. These findings suggest that despite an improvement in attitudes, the same barriers persist.

Future interventions should take into account the perceived barriers inhibiting the provision of smoking cessation care. The integration of smoking status assessment and subsequent flagging of treatment availability into electronic medical records is one prospective option to assist staff in ensuring co-ordinated and timely provision of smoking cessation care. Further, training of staff specifically in these systems and smoking cessation counselling would assist in the development of knowledge and competence. Prior research also suggests that training and delivery of educational resources has the potential to address common misconceptions and help promote more supportive smoking cessation care views among AOD staff [33, 34].

Given the consistent association between staff current tobacco use and unsupportive smoking cessation care attitudes, providing optional smoking cessation support to staff, as well as ensuring all staff regardless of smoking status are trained to deliver care, should be a particular priority. More attention needs to be given to understanding and shifting the views of smoking staff and supporting staff smoking cessation through providing free NRT and other evidenced-based treatments. Further, services without smoking ban restrictions should look towards implementing smoke-free policy and smoking cessation care protocols as this may improve the current treatment climate and culture towards smoking cessation care by AOD staff.

In regards to funding or resource allocation, the integration of evidenced-based treatment into usual care at AOD services has the potential to reduce the economic burden to clients acquiring their own treatment. In Australia, non-government and government managed AOD services are covered by a mix model of funding consisting of various activity-based and episode-of-care models. AOD services should examine whether it is possible to incorporate the purchase of NRT and other smoking cessation resources within their existing budgets or seek funding from external organisations to improve the infrastructure for delivering smoking cessation care to clients.

The findings provide valuable information for tailoring interventions to facilitate the provision of smoking cessation care in the AOD setting by identifying characteristics associated with unsupportive attitudes and the barriers impeding care. Other study strengths includes a good response rate, comparable to similar studies of AOD staff [25, 35], as well as a sample of individuals from a wide range of AOD programs; a common weakness of other AOD studies [10, 22, 36]. However, the generalisability of study findings may be limited as those services and staff who participated may hold more positive attitudes and be more interested in the delivery of smoking cessation care to AOD clients than those who did not participate. Further, the results may also be limited to the Australian AOD setting which may not generalise to other countries.

## Conclusions

Overall, AOD staff hold largely supportive smoking cessation care attitudes despite experiencing a large number of structural, resource and individual barriers to care. Characteristics associated with unsupportive smoking cessation care attitudes suggest that organisational change interventions targeting the current treatment culture, their beliefs and perceived barriers, may help facilitate greater provision of smoking cessation care to AOD clients.

## Abbreviations

AOD: Alcohol and other drug
ARIA: Accessibility/remoteness index of Australia
